# Increasing ratio of opportunistic infections associated with sunshine exposure and economic level burdening Chinese inflammatory bowel disease hospitalized patients: the first nationwide survey from 2014 to 2019

**DOI:** 10.1186/s12889-024-17635-6

**Published:** 2024-01-09

**Authors:** Runing Zhou, Ruixian Wu, Li Wang, Hong Yang

**Affiliations:** 1 Department of Gastroenterology, Peking Union Medical College Hospital, Chinese Academy of Medical Sciences and Peking Union Medical College, Beijing, 100730 China; 2 Center for Health Statistics and Information, National Health Commission, Beijing, 100044 China; 3 Department of Epidemiology and Biostatistics, School of Basic Medicine Peking Union Medical College, Institute of Basic Medical Sciences Chinese Academy of Medical Sciences, Beijing, 100005 China

**Keywords:** Inflammatory bowel disease, Opportunistic infections, China, Environmental factors

## Abstract

**Background:**

The rising prevalence of opportunistic infections (OIs) in inflammatory bowel disease (IBD) in conjunction with the use of biologics/immunosuppressive agents has garnered attention. However, there is a dearth of research on OIs in Mainland China. This study seeks to evaluate the national ratio trend of OIs in IBD and elucidate the influence of economic and climate factors on IBD patients with OIs and their outcomes.

**Methods:**

The nationwide data was obtained from the Inpatient medical record home page via the Health Statistics and Information Reporting System (HSRS). Patients diagnosed with IBD were enlisted for participation, and their demographic and clinical information, encompassing infection type, surgical procedures, and expenses, were gathered. The National Bureau of Statistics provided data on monthly sunshine exposure hours and yearly Gross Domestic Product (GDP).

**Results:**

Findings indicate that between 2014 and 2019, a total of 381,752 patients with IBD were admitted to hospitals, with 364,249 patients lacking OIs and 17,503 patients presenting with OIs. The annual proportion of OIs exhibited an upward trend, rising from 3.54% in 2014 to 4.81% in 2019. There was a significant correlation observed between individuals who identified as male, those who visited hospitals in southern regions, or those originating from areas with lower GDP or shorter sunshine exposure hours, and a higher incidence of OIs. Among patients diagnosed with either Crohn’s disease (CD) or ulcerative colitis (UC), Clostridium difficile was found to be the most prevalent infection, followed by Epstein-Barr virus and cytomegalovirus. Furthermore, the occurrence of OIs was found to be associated with an increased rate of surgical interventions in UC patients.

**Conclusions:**

The rising prevalence of OIs among hospitalized patients with IBD necessitates heightened attention towards mitigating associated risk factors, particularly among IBD patients residing in less developed regions or experiencing limited exposure to sunlight. This approach aims to minimize hospital stays and associated costs.

**Supplementary Information:**

The online version contains supplementary material available at 10.1186/s12889-024-17635-6.

## Introduction

Inflammatory bowel disease (IBD), consisting of ulcerative colitis (UC) and Crohn’s disease (CD), is a gastrointestinal disease characterized by relapsing chronic inflammation. The past few decades have witnessed dramatic increase in the incidence of IBD and the number of IBD-related hospitalizations in newly developing countries [1, 2]. The prognosis of IBD patients has improved with the advancement of medication, ranging from glucocorticoids and immunosuppressants to biological drugs. However, the increasing use of immunosuppressive and biological drugs has raised concerns about the occurrence of opportunistic infections, [3, 4] which pose a safety risk for IBD patients due to their dysregulated immune responses and malnutrition.

An opportunistic infection may be defined as a usually progressive infection by a microorganism that has limited (or no) pathogenic capacity under ordinary circumstances, but which is able to cause serious disease as a result of the predisposing effect of another disease or of its treatment [5]. Opportunistic infections can lead to frequent disease relapse and worse clinical outcomes, including higher surgery rates and mortality [6, 7]. Additionally, they are associated with longer hospitalizations, increased hospital costs, and higher overall mortality [8, 9]. Both iatrogenic factors (such as biologics or corticosteroid treatments) and personal factors, including malnutrition, high body mass index, comorbidities, active disease, living in less economically developed regions, and older age, can increase the risk of opportunistic infections [5, 10]. Therefore, studying the characteristics and risk factors associated with opportunistic infections in IBD patients may provide valuable insights into understanding the current burden of IBD in China.

Various studies have indicated a significant influence of environmental and economic factors on infection rates [11], making this relationship of great interest to scientists. The aforementioned studies demonstrated a greater risk of gastrointestinal infections among individuals with lower economic income, resulting in illness consequences such as heightened symptom severity and increased absenteeism from work or school [12]. Furthermore, the influence of climate and sunlight on infection rates was noted [13, 14]. Considering that individuals with IBD undergoing immunosuppression are immunocompromised and prone to various opportunistic infections, the precise relationship between social-environmental factors and opportunistic infections associated with IBD remains yet to be elucidated.

For this study, we gathered data on hospitalizations of Chinese IBD patients with opportunistic infections from the Health Statistics and Information Reporting System (HSRS), which serves as the largest inpatient database in mainland China. Moreover, we accessed environmental data from the China Statistical Yearbook (http://www.stats.gov.cn). The primary aim of our research was to describe the characteristics of opportunistic infections associated with IBD among Chinese hospitalized patients and examine their potential correlation with social-environmental factors.

## Materials and methods

### Sources of data

The data utilized in this study were obtained from the Inpatient Medical Record Home Page (IMRHP), which is a component of the Health Statistics and Information Reporting System (HSRS). The HSRS was established in 2008 in accordance with administrative requirements set by the China Ministry of Health. This comprehensive database encompasses both public hospitals (secondary and tertiary) and private hospitals across mainland China. The total number of registered secondary and tertiary general or specialty hospitals included in the database is 7524, accounting for approximately three quarters of all inpatient services provided in the country and with routine data quality control [15]. Inpatient-related data (demographic information, insurance state, code and Chinese wording of diagnosis, surgery/procedure, pathology result, hospitalization cost, discharge status, etc.) and hospital-related data (hospital grade, region, area, etc.) are submitted by hospitals. Diagnosis was classified both by the International Classification of Diseases, 10th revision (ICD-10) and Chinese name. To ensure data completeness and quality, the provincial and national Centers for Health Statistics and Information conduct regular examinations of item reporting within each IMRHP and assess the overall completeness of reported records. Additionally, periodic data quality control meetings, supervision activities, and inspections are conducted to uphold data integrity.

### Climate and economic information

In order to analyze the distribution pattern of IBD hospitalizations in regions with varying socioeconomic and climatic conditions, we acquired data on monthly sunshine exposure hours and annual Gross Domestic Product (GDP) from January 1st, 2014 to December 31st, 2019. These data points were sourced from the National Bureau of Statistics (http://www.stats.gov.cn/). To facilitate comparison between patient groups, we divided them into two categories using the median values of the aforementioned factors. The inadequate isolation referred to monthly sunshine exposure less than 158 h. The low GDP level referred to annual GDP less than 3,815,600 million yuan.

### IBD retrieval and validation

Initially, we conducted a thorough screening of the ICD-10 codes for all patients admitted to hospitals and recorded in the HSRS database between January 1st, 2014 and December 31st, 2019. We identified patients with a primary discharge diagnosis code for Crohn’s disease (CD) (K50.X) or ulcerative colitis (UC) (K51.X), in order to figure out the IBD-related hospitalization and avoid overt privacy breaching. This screening process yielded a total of 389,625 patients. Subsequently, we retrieved various personal variables for these patients, including demographic information such as age and gender, dates of hospital admission and discharge, primary and secondary diagnoses (up to ten in total), surgical procedures, biopsy results, and overall hospitalization costs. To ensure the accuracy of the ICD-10 codes, we conducted extensive validation procedures and cross-checked the codes with the assistance of qualified ICD-10 coders. In our analysis, we excluded cases of indeterminate colitis (K52.3), repeated admission records with matching hospital and patient IDs for each day, as well as those lacking or having incorrect demographic information. Furthermore, to be eligible for inclusion in this study, patients had to be aged 18 years or older and have undergone at least one day of hospitalization. Next, we meticulously validated the primary diagnosis codes by carefully reviewing additional variables, such as the wording of diagnoses, pathology results, and performed operations/procedures. Consequently, we excluded a total of 7873 cases due to discrepancies between the original codes and other relevant variables. Ultimately, our analysis included 224,825 cases of UC and 156,927 cases of CD.

### Definitions of variables

As ICD coding has been verified in previous studies for identifying opportunistic infection diagnoses in IBD patients, we utilized it to identify specific infections such as cytomegalovirus (CMV) (including CMV colitis, proved by biopsy results) (ICD-10: B25.901), Epstein-Barr virus (EBV) (ICD-10: B00.901), Clostridium difficile (*C.diff*) (ICD-10: A04.701), varicella-zoster virus (VZV) (ICD-10: B01) and fungal infections (ICD-10:B35-B49). The pathogens selected to be analyzed in this study were based on the Chinese evidence-based consensus on opportunistic infections in inflammatory bowel disease [16] and European Crohn’s and Colitis Organization guidelines in OIs of IBD [17]. Verification of these diagnoses relied on the ICD codes and their corresponding Chinese names extracted using regular expressions to capture Chinese characters. Additionally, although hepatitis viruses (ICD-10: B15-B19) and Mycobacterium tuberculosis (ICD-10: B90) infections are prevalent among the general population in China and may impact the clinical course of IBD, their association with IBD remains unclear and challenging to confirm within this study. Nonetheless, we included them in our analysis due to their potential influence. What’s more, specific bacterial or viral infections were not analyzed separately due to the challenges associated with their diagnosis solely through the IMRHP. The proportion of these variables served as a major evaluation of the burden imposed by IBD on hospitalized patients. Other variables related to the burden of hospitalization included the length of stay (LOS) and total charges per person per year. Other variables mentioned in the study including gastrointestinal complications (intestinal fistula, intestinal obstruction, intestinal hemorrhage, toxic megacolon, and perianal lesions) and extraintestinal manifestations (EIMs) (disorders in bones and joints, pyoderma gangrenosum and erythema nodosum, episcleritis and uveitis, gallstones and primary sclerosing cholangitis, venous thromboembolism). Both complications and EIMs were added up for one *P*-value.

### Statistical analysis

We conducted statistical analyses using the chi-square test for categorical variables and one-way analysis of variance (ANOVA) for continuous variables to compare and analyze the data. The annual percentages of OIs were calculated by dividing the number of patients presenting with such events by the total number of CD/UC patients in the database. Descriptive statistics for the length of stay (LOS) and hospital cost were presented using the median and interquartile range (IQR). Hospitalization cost represented the overall cost incurred during a patient’s hospital stay. Furthermore, we determined the annual percentage change (APC) of complication and surgery proportions using a log-linear model, alongside 95% confidence intervals (CIs). Our statistical analyses were performed using SAS 9.4 (SAS Institute, Cary, NC, USA) and Joinpoint version 4.7 (National Cancer Institute, Calverton, MD, USA). All statistical tests were two-tailed, and a significance level of *P* < 0.05 was considered indicative of statistical significance.

## Results

### Baseline characteristics of the IBD hospitalized patients with or without opportunistic infections

Following the completion of data cleaning procedures, we successfully included the records of 224,825 patients with UC code (K51) and 156,927 patients with CD code (K50) from the hospitalization summary reports database between 2014 and 2019. Among all IBD hospitalized patients, a total of 17,503 individuals suffered from at least one opportunistic infection, constituting 4.46% of the total population (Table 1). Notably, the ratio of men (11,654, 5.12%) and patients admitted to southern hospitals (12,840, 4.92%) was higher when compared to women (5,849, 3.79%) or patients in northern hospitals (4,663, 3.86%) (*P* = 0.000). Patients with infections tended to stay longer (9 days vs. 7 days, *P* = 0.000) and incurred higher costs during their hospitalization (*P* = 0.000). Additionally, among various levels of hospitals, tertiary hospitals exhibited the highest proportion of infectious IBD patients (5.13%).

**Table 1:** Baseline information of IBD hospitalized patients with or without OIs

	without OIs (*n* = 364,249)	with OIs (*n* = 17,503)	***P*** value
Primary disease (%)			0.000
UC	215,836 (95.40%)	8989 (3.40%)	
CD	148,413 (94.57%)	8514 (5.43%)	
Gender (%)			0.000
Male	215,880 (94.88%)	11,654 (5.12%)	
Female	148,369(96.21%)	5849 (3.79%)	
Region (%)			0.000
North	116,097 (96.14%)	4663 (3.86%)	
South	248,152 (95.08%)	12,840 (4.92%)	
Average age at administration (year old)	43.46 ± 18.11	43.28 ± 15.99	0.173
Median length of hospitalization (day)	7 [3, 12]	9 [4, 14]	0.000
Median cost of hospitalization (yuan)	5797.12 [2831.64,10642.37]	8485.16 [4210.19, 15817.00]	0.000
Hospital level (%)			0.000
Primary	3201 (98.49%)	49 (1.51%)	
Secondary	69,616 (97.35%)	1896 (2.65%)	
Tertiary	283,842 (94.87%)	15,336 (5.13%)	
Unknown	6606 (96.83%)	216 (3.17%)	
Gastrointestinal complications (%)			0.000
No	335,568 (92.13%)	15,516 (88.65%)	
Yes	28,681 (7.87%)	1987 (11.35%)	
Perianal lesion (%)			0.000
No	348,902 (95.79%)	16,499 (94.26%)	
Yes	15,347 (4.21%)	1004 (5.74%)	
Extraintestinal manifestations (%)			0.000
No	341,396 (93.73%)	158,151 (90.36%)	
Yes	22,853 (6.27%)	1688 (9.64%)	
Gastrointestinal surgery (%)			0.003
No	359,185 (98.61%)	17,212 (98.34%)	
Yes	5064 (1.39%)	291 (1.66%)	
Way of discharge (%)			0.000
Regular discharge	336,893 (92.49%)	16,136 (92.19%)	
Regular transfer	2403 (0.66%)	124 (0.71%)	
Transfer to nursery	702 (0.19%)	36 (0.21%)	
Non-regular discharge	9845 (2.70%)	665 (3.80%)	
Death	175 (0.05%)	10 (0.06%)	
Unknown	14,231 (3.91%)	532 (3.04%)	

**Table 2:** Social/ environmental factors and characteristic of IBD patients

	without OIs (*n* = 364,249)	with OIs (*n* = 17,503)	***P*** value
GDP (100 million yuan)			0.001
<38,156	180,680 (95.30%)	8915 (4.70%)	
>=38,156	183,569 (95.53%)	8588 (4.47%)	
Monthly sunshine hours (hours)			0.000
<158	182,397 (95.19%)	9221 (4.81%)	
>=158	181,852 (95.64%)	8282 (4.36%)	
Hospital level (%)			0.000
Primary	3201 (98.49%)	49 (1.51%)	
Secondary	69,616 (97.35%)	1896 (2.65%)	
Tertiary	283,842 (94.87%)	15,336 (5.13%)	
Unknown	6606 (96.84%)	216 (3.17%)	

The incidence of gastrointestinal complications, perianal lesions, extra-intestinal manifestations, and gastrointestinal surgeries exhibited a significantly higher rate among individuals with opportunistic infections (*P* < 0.05). However, there was no difference in the mortality ratio between the two groups, which stood at 0.05%.

**Fig. 1:**
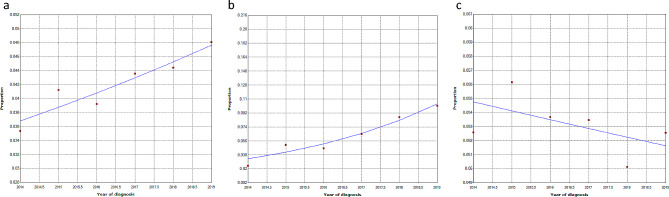
The annual percentage change of OIs in (a) all IBD patients; (b) UC patients; (c) CD patients

Among the total IBD hospitalized patients, the UC associated with OI were in the significantly increasing trend, with the annual percentage change of 5.3% (2.6-8.1%) (Fig. 1a) and 25.8% (14.7-38%) (Fig. 1b) respectively (*P* < 0.05). Conversely, there was a declining trend in CD cases associated with opportunistic infections, indicating a percentage change of 1.2% (with a confidence interval of -3.6%-1.3%) (*P* = 0.3) (Fig. 1c).

### Social/ environmental factors and clinical outcomes of IBD patients with or without opportunistic infections

Among all hospitalized IBD patients, it was found that individuals who visited hospitals in regions with lower GDP had a higher proportion of infections (4.70% vs. 4.47%, *P* = 0.001). Additionally, inadequate isolation increased the risk of opportunistic infections (*P* = 0.000), which is consistent with the observation of a higher infection rate in the southern regions—known for their hotter and more humid climate—compared to the northern regions (Table 2).

Among all hospitalized CD patients, a total of 8,514 individuals (5.43%) experienced at least one type of opportunistic infection (Table 3). Notably, men (5.72%) and patients who sought care at tertiary hospitals (5.62%) exhibited a higher ratio of infections (*P* = 0.000). There was more CD inpatients with infections in hospitals located in the northern than in the southern regions (*P* = 0.011). Furthermore, patients with infections were found to be statistically older and had longer hospital stays, resulting in increased healthcare costs (*P* = 0.000, respectively). Our assessment indicated that the proportion of infected patients reached its peak in regions with lower GDP and shorter sunshine hours (*P* < 0.05, respectively).

**Table 3:** Social/ environmental factors and characteristic of CD patients

	Opportunistic infections		
	No (*n* = 148,413)	Yes (*n* = 8514)	***P*** value
Gender			0.000
Male	99,393 (94.28%)	6032 (5.72%)	
Female	49,020 (95.18%)	2482 (4.82%)	
Region			0.011
North	18,370 (94.19%)	1134 (5.81%)	
South	130,043 (94.63%)	7380 (5.37%)	
Average age (year old)	32.80 ± 14.79	36.87 ± 13.81	0.000
Median length of stay (day)	3 [1, 9]	6 [2, 12]	0.000
Median cost of stay (yuan)	5496.00 [1174.44,13480.21]	8623.28 [2813.99,16485.28]	0.000
GDP (100 million yuan)			0.040
<38,156	83,955 (94.47%)	4913 (5.53%)	
>=38,156	64,458 (94.70%)	3601 (5.29%)	
Monthly sunshine hours (hours)			0.001
<158	82,469 (94.41%)	4885 (5.59%)	
>=158	65,944 (94.78%)	3629 (5.22%)	
Hospital level (%)			0.000
Primary	282 (95.92%)	12 (4.08%)	
Secondary	11,029 (96.72%)	374 (3.28%)	
Tertiary	135,072 (94.38%)	8042 (5.62%)	
Unknown	1923 (95.77%)	85 (4.23%)	

Shifting our focus to UC patients, it was discovered that out of the total cases, 8,989 individuals (4.00%) suffered from at least one type of opportunistic infection (Table 4). Among this group, men (4.60%) and those who received medical care in southern hospitals (4.42%) demonstrated a higher infection ratio (*P* = 0.000). Interestingly, unlike CD patients, UC patients with infections were statistically younger (49.35 ± 15.54 vs. 50.80 ± 16.47), yet still experienced extended hospital stays and higher healthcare expenses (*P* = 0.000, respectively). The peak proportion of infected patients was observed in regions with lower levels of sunshine exposure (*P* = 0.000, respectively). Notably, there was no statistical difference observed between regions with low and high GDP (*P* = 0.588).

**Table 4:** Social/ environmental factors and characteristic of UC patients

	Opportunistic infections		
	No (*n* = 215,836)	Yes (*n* = 8989)	***P*** value
Gender			0.000
Male	116,487 (95.40%)	5622 (4.60%)	
Female	99,349 (96.72%)	3367 (3.28%)	
Region			0.000
North	97,727 (96.51%)	3529 (3.49%)	
South	118,109 (95.58%)	5460 (4.42%)	
Average age (year old)	50.80 ± 16.47	49.35 ± 15.54	0.000
Median length of stay (day)	9 [6, 13]	10 [7, 16]	0.000
Median cost of stay (yuan)	5890.59 [3619.10,9492.35]	8362.97 [4955.17,14945.47]	0.000
GDP (100 million yuan)			0.588
<38,156	119,111 (95.98%)	4987 (4.02%)	
>=38,156	96,725 (96.03%)	4002 (3.97%)	
Monthly sunshine hours (hours)			0.000
<158	99,928 (95.84%)	4336 (4.16%)	
>=158	115,908 (96.14%)	4653 (3.86%)	
Hospital level (%)			0.000
Primary	2919 (98.75%)	37 (1.25%)	
Secondary	58,587 (97.47%)	1522 (2.53%)	
Tertiary	148,770 (95.33%)	7294 (4.67%)	
Unknown	4683 (97.28%)	131 (2.72%)	

### Ratio, age and gender distribution of opportunistic infections with different pathogens in IBD hospitalized patients

We conducted an analysis to estimate the prevalence of five common pathogens in opportunistic infections associated with IBD among hospitalized patients (Supplementary Table S1). Among Chinese IBD patients, the highest prevalence was found in hepatitis virus infections (CD: 6.36%, UC: 2.10%, *P* < 0.000). Subsequently, Mycobacterium tuberculosis (1.66%) and *C.diff* (0.36%) ranked next for CD patients, while this order was reversed for UC patients (*C.diff*: 1.85%, Mycobacterium tuberculosis: 0.87%). EBV and CMV infections were also identified but with lower prevalence rates. When comparing CD and UC patients, the prevalence ratios of *C.diff*, CMV, EBV, VZV and fungal infections were significantly higher in UC than in CD (*P* < 0.000), while the ratios of Mycobacterium tuberculosis and hepatitis virus infections appeared to be higher in CD (*P* < 0.000).

The age distribution among patients with different pathogen infections exhibited notable variations. The overall infection ratio increased with age, suggesting that older hospitalized patients faced a higher risk. However, for CMV, EBV, and *C.diff* infections, the ratio decreased with age in both CD and UC patients (Supplementary Table S2-1, S2-2). These infections were most prevalent among CD patients younger than 16 years old and UC patients younger than 34 years old. On the other hand, Mycobacterium tuberculosis and hepatitis virus infections were predominantly observed among CD patients older than 40 years old (*P* < 0.000). In UC patients, the highest ratio of Mycobacterium tuberculosis infections was recorded in the oldest age group (≥ 68 years old), while hepatitis virus infections were most common among individuals aged 35–51 years old. Unexpectedly, the highest overall infection ratio was found among patients younger than 51 years old.

The prevalence of pathogen infections varied across genders among patients (Supplementary Table S3-1, S3-2). In CD patients, a significantly higher ratio of CMV, *C.diff* and VZV infections was observed in females (*P* < 0.05), while the ratio of Mycobacterium tuberculosis or hepatitis virus infections was higher in males (*P* < 0.001). No statistical difference was found in EBV and fungal infections (*P* = 0.096). Conversely, in UC patients, the ratio of infections for all the aforementioned pathogens, except for *C.diff* and VZV, was significantly higher in males (*P* = 0.522 and 0.323 respectively).

### Surgery ratio and mortality in IBD hospitalized patients with opportunistic infections

The rate of surgical interventions was significantly higher in UC patients with opportunistic infections (0.86% vs. 1.26%, *P* < 0.000) (Supplementary Table S4). However, in CD patients, there was no significant difference between those with and without opportunistic infections regarding surgical procedures (2.16% vs. 2.09%, *P* = 0.698).

Furthermore, there was no statistical difference in mortality rates between patients with or without opportunistic infections, both in CD and UC groups (CD: 0.06 vs. 0.07%, *P* = 0.648; UC: 0.04% in both group, *P* = 0.784) (Supplementary Table S5), which is similar to the limited report in China [18].

## Discussion

This study represents the first comprehensive investigation on the characteristics of opportunistic infections in IBD hospitalized patients across mainland China, covering a significant temporal and spatial range. The key findings can be summarized as follows. Firstly, OIs are a common occurrence during the course of IBD, with a total of 17,503 individuals suffered from at least one opportunistic infection, constituting 4.46% between 2014 and 2019 in China. Secondly, the presence of opportunistic infections significantly exacerbates the burden on both individuals and the national healthcare system. There are four main aspects to consider: Firstly, the incidence rate of IBD with opportunistic infections is increasing year by year, particularly among UC patients. The ratio change in IBD is 5.3%, while in UC it is 25.8% and in CD it is 1.2%. Additionally, opportunistic infections not only lead to prolonged hospital stays and increased healthcare expenditures for patients, but also raise the risk of gastrointestinal complications and the need for surgery. Thirdly, the most common pathogens associated with opportunistic infections in both UC and CD patients were *C. diff*, followed by EBV and CMV. Moreover, there was a higher proportion of males among IBD patients with opportunistic infections, and younger hospitalized patients exhibited a higher prevalence of *C. diff*, CMV, and EBV infections. Lastly, lower GDP regions (indicating less economic development) and shorter exposure to sunlight were associated with an increased likelihood of opportunistic infections in IBD patients.

Firstly, it was observed that opportunistic infections were prevalent among IBD inpatients in China, and their incidence has been consistently increasing over time. This finding supports the current understanding that the ratio of opportunistic infections among IBD patients is on the rise. A nationwide survey on intestinal infections in IBD patients conducted in the United States showed a significant increase in incidence rates from 18.0 to 47.4/1000 CD hospitalizations and 39.5-110.1/1000 UC hospitalizations (*P*trend < 0.01) during the period spanning from 1998 to 2014 [8]. A comprehensive nationwide study conducted in the United States between 1999 and 2018 reported a prevalence of opportunistic infections in CD patients at 17.8% and in UC patients at 19.2% [19]. Despite the current lower prevalence of IBD-associated opportunistic infections in China compared to countries with a higher incidence of IBD, it is imperative to prioritize the management of opportunistic infections. This becomes even more significant given the growing number of IBD patients and the aging population in China.

More specifically, UC patients exhibited a statistically higher ratio of *C.diff*, CMV, and EBV infections compared to other pathogens. Notably, *C.diff* infections accounted for the largest proportion among all the opportunistic infections included in the study. Moreover, previous research has consistently shown a higher prevalence of *C.diff* infections among UC patients when compared to CD patients. In a retrospective case-control study conducted in China among hospitalized patients with IBD and *C.diff* infections, the estimated prevalence of C.diff infections was reported to be 6.06% for CD patients and 7.41% for UC patients [20]. In Canada, the prevalence of *C.diff* infection was observed to be 3.73% among UC patients and 1.09% among CD patients [12]. From 1998 to 2014, there was a notable rise in the incidence of *C.diff* infections leading to hospitalization among UC patients in the United States, almost doubling over that period. Notably, *C.diff* infection was associated with a significantly higher mortality rate among patients with UC (OR = 3.79, 95% CI 2.84–5.06), whereas this association was not observed in CD patients (OR 1.66, 95% CI 0.75–3.66). Moreover, *C.diff* infections were also associated with longer hospital stays and higher average hospital charges. In relation to CMV infection, a prior study conducted in China demonstrated that the prevalence of anti-CMV IgG positivity among IBD patients was 76.11%, surpassing the rate observed in healthy controls (50.69%). Furthermore, the study indicated that pancolitis could enhance CMV proliferation [22]. Regarding CMV colitis, a recent multicenter study carried out in China revealed a prevalence of 3.1% in UC patients and 0.8% in CD patients, [23], which is consistent with several previous reports from Asia [24]. In terms of EBV infection, earlier single-center studies conducted in China have revealed a broad range of prevalence rates for EBV in the intestinal mucosa of patients, ranging from 33–79.4% [25–28]. These studies have additionally established a connection between EBV presence and clinical disease activities. Notably, the observed prevalence rates were considerably higher than those observed in our current study. In our study, we noted that the proportions of *C.diff*, EBV, and CMV infections were comparatively lower when compared to previous studies conducted in China. This disparity can be attributed to several factors. Firstly, other studies may have predominantly included more severe cases, while our study encompassed a diverse representation of patients across varying severity levels. Additionally, the underestimation of pathogen detection could be due to the limited availability of laboratory equipment in certain regions of China.

Our study revealed that IBD-associated opportunistic infections had a substantial influence on both the duration and expenses related to hospital stays. Furthermore, these infections were identified as a significant risk factor for poor outcomes, especially in relation to surgery rates among UC patients. Notably, the ratio of emergent operations could potentially rise as high as 50% in UC patients with opportunistic infections [29]. In the era of biologics, although several meta-analyses have shown the efficacy of anti-TNF biologics in significantly reducing hospitalization rates by approximately 50% and surgery rates by 33–77% in IBD patients, [30] it is essential to recognize the potential adverse effects of these medications in terms of triggering opportunistic infections. Infected patients typically face more intricate gastrointestinal inflammation and are prone to being less responsive to corticosteroid treatment, necessitating an increased likelihood of therapy escalation [31–33]. Given that the existing data in China mainly stem from retrospective studies conducted in single centers, there is a clear need for future prospective multicenter studies to comprehensively evaluate the influence of opportunistic infections on IBD outcomes. In the meantime, it is vital to emphasize regular screening, early detection, and appropriate treatment of opportunistic infections. By doing so, we can not only enhance patient outcomes but also minimize avoidable costs associated with their management.

Regions with lower GDP tend to have a higher proportion of patients with IBD-associated opportunistic infections. Numerous studies have underscored the crucial role of socioeconomic factors, including poverty, overcrowding, and poor nutrition, in the genesis of opportunistic infections. This relationship extends throughout different diseases, from historical cholera outbreaks to the current landscape of tuberculosis [34–36]. Gastrointestinal infections are commonly caused by factors such as contaminated food or water, environmental conditions, and contact with animals. It has been observed that in high-income countries like the UK, where a significant portion of the population enjoys access to healthcare, sanitation facilities, and clean water, the mortality rate due to gastrointestinal infections remains relatively low [37]. Given the negative impact of underdeveloped economies on the occurrence of infections and the potential increase in the number of IBD patients in less developed regions, [38] it is essential to recognize the heightened burden of IBD-associated opportunistic infections in these areas. Additionally, it is important to note that patients in these regions face challenges in accessing timely diagnosis and treatment, often due to limited healthcare resources and the absence of adequate medical insurance coverage, which can contribute to worse outcomes.

Additionally, we performed an examination of the relationship between meteorological factors and the risk of opportunistic infections. In both CD and UC patient groups, we discovered that decreased sunlight exposure was associated with an increased likelihood of opportunistic infections. Specifically, among CD patients, we noted a higher proportion of hospitalized individuals with opportunistic infections in Northern China, indicating regions located at higher latitudes. Conversely, among UC patients, opportunistic infections were more prevalent in Southern China, despite exhibiting a negative correlation with sunlight exposure. Throughout history, people have long acknowledged the vital role of sunlight as an important defense against infections [39–41]. Insufficient sunlight exposure has demonstrated a robust association with the activation or dissemination of various pathogens, such as respiratory syncytial virus and, notably, COVID-19 [13, 42]. Sunlight and ultraviolet radiation are known to exert dual effects in combating infections. On one hand, they can effectively eradicate pathogens, including *C.diff*. On the other hand, they play a role in bolstering an individual’s resistance to infections by regulating the biological rhythms of specific anti-inflammatory molecules, such as the Vitamin D receptor [43, 44]. Findings from a rigorously conducted randomized controlled trial revealed that the administration of oral vitamin D supplementation effectively decreased the likelihood of upper respiratory infections in individuals diagnosed with IBD [45]. Drawing upon the case of tuberculosis (TB) treatment, [46] it is important to highlight that interventions involving vitamin D, phototherapy, and sunlight exposure have consistently exhibited their capacity to effectively eliminate TB infections, culminating in their recognition through the prestigious Nobel Prize. Translating this knowledge into clinical practice, it becomes imperative to integrate routine monitoring of serum vitamin D levels and administer appropriate supplementation to IBD patients, especially those residing in geographical regions characterized by higher latitudes or who face limitations in accessing regular sunlight exposure (e.g., the elderly, disabled individuals). By implementing these interventions, there exists a promising opportunity to achieve both cost-effective and safe methods of reducing the incidence of opportunistic infections.

This comprehensive and nationwide database greatly strengthens the statistical power to identify statistically significant associations between IBD-associated opportunistic infections and vital clinical outcomes, including colectomy rates and mortality. The broad coverage encompassing diverse geographic regions and hospitals contributes to the robust generalizability of these findings. Furthermore, the incorporation of demographic and social-environmental data enables a stratified analysis that effectively controls for various potential confounding variables. Since the travel history and immigration harder the differentiation of infectious colitis and IBD as well as increasing the possibility for pathogens prevail, [46] we suggest that doctors should always take infectious diseases into consideration when they meet patients from areas with low GDP and shorter sun shine hours.

This study is subject to several limitations. Firstly, the reliance on ICD-10 codes for case retrieval from the HSRS database limits the availability of detailed clinical or laboratory data on opportunistic infections and the diagnosis of IBD. Consequently, some opportunistic pathogens were excluded from our analysis due to the absence of confirmatory testing results. To ensure the accuracy of the IBD and OI diagnoses, we employed cross-checking of information, examining not only the ICD codes but also variables related to the descriptions and wordings used in the diagnoses. Additionally, we reviewed other supporting evidence such as pathology results from biopsies and surgical operations. Secondly, we lacked data on the specific immunosuppressive agents prescribed to hospitalized IBD patients, impeding a comprehensive assessment of the association between IBD and opportunistic infections. Nevertheless, it is indeed important to assess the correlation between advanced medical modalities and opportunistic infections and a more comprehensive database is needed for the aforementioned relationship analysis. Thirdly, the HSRS database does not provide information on the specific disease behavior and location within the spectrum of CD or UC, despite these factors being known to influence the incidence of opportunistic infections. At last, specific bacterial infections or viral infections (pneumonia, meningitis) were excluded in this study due to the difficulty to withdraw the diagnosis only from the patients’ home page. To address these limitations and gather more nuanced insights into the disease, future multi-center studies are warranted, allowing for the inclusion of detailed disease profiles and assessment of associated risk factors.

## Conclusions

The burden of opportunistic infections associated with IBD in China shows an increasing trend, particularly impacting elderly patients and regions with lower levels of economic development. Notably, this burden has significant implications for the intervention rates of surgery in patients with UC. Consequently, hospitalizations of IBD patients, particularly those complicated by opportunistic infections, present an ongoing and growing challenge for the Chinese healthcare system and society at large. This study represents the most extensive investigation conducted thus far regarding the clinical manifestations, outcomes, and associated risk factors of IBD-related hospitalizations in mainland China. The insights gained from this study provide a crucial groundwork for refining clinical practices and alleviating the burden imposed by IBD in the country.

### Electronic supplementary material

Below is the link to the electronic supplementary material.

Supplementary Material 1

## Data Availability

The data that support the findings of this study are available from National Health Commission of the People’s Republic of China but restrictions apply to the availability of these data, which were used under license for the current study, and so are not publicly available. Data are however available from the authors upon reasonable request and with permission of National Health Commission of the People’s Republic of China.

## Abbreviations

IBD: Inflammatory bowel disease
UC: Ulcerative colitis
CD: Crohn’s disease
HSRS: Health Statistics and Information Reporting System
IMRHP: Inpatient medical record home page
ICD-10: International Classification of Diseases, 10th revision
GDP: Gross Domestic Product
CMV: Cytomegalovirus
EBV: Epstein-Barr virus
*C.diff*: Clostridium difficile
VZV: Varicella-zoster virus
EIM: Extraintestinal manifestations
LOS: Length of stay
ANOVA: One-way Analysis of Variance
OIs: Opportunistic infections
IQR: Interquartile range
APC: Annual percentage of change
CIs: Confidential intervals
