# Supplementary material for: Increasing ratio of opportunistic infections associated with sunshine exposure and economic level burdening Chinese inflammatory bowel disease hospitalized patients: the first nationwide survey from 2014 to 2019
Source: BMC Public Health. 2024 Jan 9;24:133. doi: 10.1186/s12889-024-17635-6 (PMC10777555; doi:10.1186/s12889-024-17635-6)
Supplement: Supplementary file 1 — Supplementary Material 1 [file 12889_2024_17635_MOESM1_ESM.docx]

**Additional material:**

- File name: 【BMC】Supplementary Table S1-S5
- File format: docx.
- Title of data:
- Supplementary Table S1 Ratio of opportunistic infections with different pathogens in IBD
- Supplementary Table S2-1 Age distribution of opportunistic infections with different pathogens in CD hospitalized patients
- Supplementary Table S2-2 Age distribution of opportunistic infections with different pathogens in UC hospitalized patients
- Supplementary Table S3-1 Gender distribution of opportunistic infections with different pathogens in CD hospitalized
- Supplementary Table S3-2 Gender distribution of opportunistic infections with different pathogens in UC hospitalized
- Supplementary Table S4 Surgery ratio in IBD hospitalized patients with opportunistic infections
- Supplementary Table S5 Mortality in IBD hospitalized patients with opportunistic infections
- Description of data: Supplementary tables of the research

Supplementary Table S1 Ratio of opportunistic infections with different pathogens in IBD

|  | CD (n=156927) | UC (n=224825) | *P* value |
| --- | --- | --- | --- |
| C.diff |  |  | 0.000 |
| No | 156360 (99.64%) | 220674 (98.15%) |  |
| Yes | 567 (0.36%) | 4151 (1.85%) |  |
| CMV |  |  | 0.000 |
| No | 156750 (99.89%) | 224030 (99.65%) |  |
| Yes | 177 (0.11%) | 795 (0.35%) |  |
| EBV |  |  | 0.000 |
| No | 156579(99.78%) | 223959 (99.61%) |  |
| Yes | 348 (0.22%) | 866 (0.39%) |  |
| TB |  |  | 0.000 |
| No | 154324 (98.34%) | 222859 (99.13%) |  |
| Yes | 2603 (1.66%) | 1966 (0.87%) |  |
| Hepatitis virus |  |  | 0.000 |
| No | 146969 (93.65%) | 220097 (97.90%) |  |
| Yes | 9958 (6.36%) | 4728 (2.10%) |  |
| VZV |  |  | 0.000 |
| No | 156841 (99.95%) | 224561 (99.88%) |  |
| Yes | 86 (0.05%) | 264 (0.12%) |  |
| Fungal infections |  |  | 0.000 |
| No | 156719 (99.87%) | 224340 (99.78%) |  |
| Yes | 208 (0.13%) | 485 (0.22%) |  |

Supplementary Table S2-1 Age distribution of opportunistic infections with different pathogens in CD hospitalized patients

|  | | <16 (N=18142) | | | | 16-40 (N=94542) | | | | >40 (N=44243) | | *P* value | |  |
| --- | --- | --- | --- | --- | --- | --- | --- | --- | --- | --- | --- | --- | --- | --- |
| C.diff | |  | | | |  | | | |  | | 0.000 | |  |
| No | | 14402 (99.61%) | | | | 100835 (99.92%) | | | | 41538 (99.96%) | |  | |  |
| Yes | | 56 (0.39%) | | | | 78 (0.08%) | | | | 18 (0.04%) | |  | |  |
| CMV | |  | | | |  | | | |  | | 0.002 | |  |
| No | | 14429 (99.80%) | | | | 100815 (99.90%) | | | | 41506 (99.88%) | |  | |  |
| Yes | | 29 (0.20%) | | | | 98 (0.10%) | | | | 50 (0.12%) | |  | |  |
| EBV | |  | | | |  | | | |  | | 0.000 | |  |
| No | | 14384 (99.49%) | | | | 100725 (99.81%) | | | | 41470 (99.79%) | |  | |  |
| Yes | | 74 (0.51%) | | | | 188 (0.19%) | | | | 86 (0.21%) | |  | |  |
| TB | |  | | | |  | | | |  | | 0.000 | |  |
| No | | 14284 (98.80%) | | | | 99524 (98.62%) | | | | 40516 (97.50%) | |  | |  |
| Yes | | 174 (1.20%) | | | | 1389 (1.38%) | | | | 1040 (2.50%) | |  | |  |
| Hepatitis virus | | | |  | | | |  | | | | 0.000 | |  |
| No | | 14423 (99.76%) | | | | 97639 (96.76%) | | | | 39635 (95.38%) | |  | |  |
| Yes | | 35 (0.24%） | | | | 3274 （3.24%） | | | | 1921 （4.62%） | |  | |  |
| VZV | | | | |  | | | |  | | | | 0.000 | |
| No | | | 18142 (100.00%) | | | | 94505 (99.96%) | | | | 44194 (99.89%) | |  | |
| Yes | | | 0 (0%) | | | | 37 (0.04%) | | | | 49 (0.11%) | |  | |
| Fungal infections | | | | |  | | | |  | | | | 0.000 | |
| No | | | 18108 (99.81%) | | | | 94456 (99.91%) | | | | 44155 (99.80%) | |  | |
| Yes | | | 34 (0.19%) | | | | 86 (0.09%） | | | | 88 (0.20%) | |  | |
| Total | |  | | | |  | | | |  | | 0.000 | |  |
| No | | 17671 (97.40%) | | | | 89822 (95.01%) | | | | 40920 (92.49%) | |  | |  |
| Yes | | 471 (2.60%) | | | | 4720 (4.99%) | | | | 3323 (7.51%) | |  | |  |

Supplementary Table S2-2 Age distribution of opportunistic infections with different pathogens in UC hospitalized patients

|  | ≤34 (N=40733) | 35-51 (N=69794) | 52-67 (N=79721) | ≥68 (N=34577) | *P* value |
| --- | --- | --- | --- | --- | --- |
| C.diff |  |  |  |  | 0.000 |
| No | 40601 (99.68%) | 64182 (99.83%) | 85086 (99.84%) | 34541 (99.90%) |  |
| Yes | 132 (0.32%) | 108 (0.17%) | 139 (0.16%) | 36 (0.10%) |  |
| CMV |  |  |  |  | 0.000 |
| No | 40493 (99.41%) | 64065 (99.65%) | 84957 (99.69%) | 34515 (99.82%) |  |
| Yes | 240 (0.59%) | 225 (0.35%) | 268 (0.31%) | 62 (0.18%) |  |
| EBV |  |  |  |  | 0.000 |
| No | 40479 (99.38%) | 64036 (99.60%) | 84927 (99.65%) | 34517 (99.83%) |  |
| Yes | 254 (0.62%) | 254 (0.40%) | 298 (0.35%) | 60 (0.17%) |  |
| TB |  |  |  |  | 0.000 |
| No | 40451 (99.31%) | 63744 (99.15%) | 84481 (99.13%) | 34183 (98.86%) |  |
| Yes | 282 (0.69%) | 546 (0.85%) | 744 (0.87%) | 394 (1.14%) |  |
| Hepatitis virus | | |  |  | 0.000 |
| No | 39957 (98.09%) | 62437 (97.12%) | 83560 (98.05%) | 34143 (98.74%) |  |
| Yes | 776 (1.91%) | 1853 (2.88%) | 1665 (1.95%) | 434 (1.26%) |  |
| VZV |  |  |  |  | 0.000 |
| No | 40711 (99.95%) | 69747 (99.93%) | 79611 (99.86%) | 34492 (99.75%) |  |
| Yes | 22 (0.05%) | 47 (0.07%) | 110 (0.14%) | 85 (0.25%) |  |
| Fungal infections |  |  | 0.000 |  | 0.421 |
| No | 40643 (99.78%) | 69659(99.81%) | 79535 (99.77%) | 34503 (99.79%) |  |
| Yes | 90 (0.22%) | 135 (0.19%) | 186 (0.23%) | 74 (0.21%) |  |
| Total |  |  |  |  | 0.000 |
| No | 39040 (95.84%) | 66575 (95.39%) | 76741 (96.26%) | 33480 (96.83%) |  |
| Yes | 1693 (4.16%) | 3219 (4.61%) | 2980 (3.74%) | 1097 (3.17%) |  |

Supplementary Table S3-1 Gender distribution of opportunistic infections with different pathogens in CD hospitalized

|  | Male (N=105425) | Female (N=51502) | *P* value |
| --- | --- | --- | --- |
| C.diff |  |  | 0.029 |
| No | 105336 (99.92%) | 51439 (99.88%) |  |
| Yes | 89 (0.08%) | 63 (0.12%) |  |
| CMV |  |  | 0.019 |
| No | 105321 (99.90%) | 51429 (99.86%) |  |
| Yes | 104 (0.10%) | 73 (0.14%) |  |
| EBV |  |  | 0.096 |
| No | 105206 (99.79%) | 51373 (99.75%) |  |
| Yes | 219 (0.21%) | 129 (0.25%) |  |
| TB |  |  | 0.001 |
| No | 103595 (98.26%) | 50729 (98.50%) |  |
| Yes | 1830 (1.74%) | 773 (1.50%) |  |
| Hepatitis virus |  |  | 0.000 |
| No | 101634 (96.40%) | 50063 (97.21%) |  |
| Yes | 3791 (3.60%) | 1439 (2.79%) |  |
| VZV |  |  | 0.027 |
| No | 105377 (99.95%) | 51464 (99.93%) |  |
| Yes | 48 (0.05%) | 38(0.07%) |  |
| Fungal infections |  |  | 0.715 |
| No | 105288 (99.87%) | 51431 (99.86%) |  |
| Yes | 1371 (0.13%) | 71 (0.14%) |  |

Supplementary Table S3-2 Gender distribution of opportunistic infections with different pathogens in UC hospitalized

|  | Male (N=122109) | Female (N=102716) | *P* value |
| --- | --- | --- | --- |
| C.diff |  |  | 0.522 |
| No | 121877 (99.81%) | 102533 (99.82%) |  |
| Yes | 232 (0.19%) | 183 (0.18%) |  |
| CMV |  |  | 0.000 |
| No | 121621 (99.60%) | 102409 (99.70%) |  |
| Yes | 488 (0.40%) | 307 (0.30%) |  |
| EBV |  |  | 0.000 |
| No | 121563 (99.55%) | 102396 (99.69%) |  |
| Yes | 546 (0.45%) | 320 (0.31%) |  |
| TB |  |  | 0.000 |
| No | 120783 (98.91%) | 102076 (99.38%) |  |
| Yes | 1326 (1.09%) | 640 (0.62%) |  |
| Hepatitis virus | |  | 0.000 |
| No | 119146 (97.57%) | 100951 (98.28%) |  |
| Yes | 2963 (2.43%) | 1765 (1.72%) |  |
| VZV |  |  | 0.323 |
| No | 121974 (99.89%) | 102587 (99.87%) |  |
| Yes | 135 (0.11%) | 129 (0.13%) |  |
| Fungal infections |  |  | 0.062 |
| No | 121825 (99.77%) | 102515 (99.80%) |  |
| Yes | 284 (0.23%) | 201 (0.20%) |  |

Supplementary Table S4 Surgery ratio in IBD hospitalized patients with opportunistic infections

|  | CD | |  |  | UC | |  |
| --- | --- | --- | --- | --- | --- | --- | --- |
|  | No (n=148413) | Yes (n=8514) | *P* value |  | No (n=215836) | Yes (n=8989) | *P* value |
| Surgery |  |  | 0.698 |  |  |  | 0.000 |
| No | 145210 (97.84%) | 8336 (97.91%) |  |  | 213975 (99.14%) | 8876 (98.74%) |  |
| Yes | 3203 (2.16%) | 178 (2.09%) |  |  | 1861 (0.86%) | 113 (1.26%) |  |

Supplementary Table S5 Mortality in IBD hospitalized patients with opportunistic infections

|  | CD | |  |  | UC | |  |
| --- | --- | --- | --- | --- | --- | --- | --- |
|  | No (n=148413) | Yes (n=8514) | *P* value |  | No (n=215836) | Yes (n=8989) | *P* value |
| Mortality |  |  | 0.648 |  |  |  | 0.784 |
| No | 148324 (99.94%) | 8508 (99.93%) |  |  | 215750 (99.96%) | 8985 (99.95%) |  |
| Yes | 89 (0.06%) | 6 (0.07%) |  |  | 86 (0.04%) | 4 (0.04%) |  |
